# Evaluating Stigmatization Toward Mental Illnesses Among Resident Physicians

**DOI:** 10.7759/cureus.61648

**Published:** 2024-06-04

**Authors:** Jamon Hemingway, Justin Kunz, David Draney, David E Martin, Suporn Sukpraprut-Braaten

**Affiliations:** 1 Psychiatry, Unity Health, Searcy, USA; 2 Medicine, Unity Health, Searcy, USA; 3 Medicine, Kansas City University, Kansas City, USA

**Keywords:** schizophrenia, amiq, bias, resident physicians, stigmatization, mental illness, stigma

## Abstract

Background

Individuals with mental illness are stigmatized by peers in societal, workplace, and healthcare settings. The stigmatization individuals experience from healthcare providers is well documented and pervasive, often being a detriment to the quality of healthcare these individuals receive.

Objective

Recognizing and addressing stigmatization toward individuals with mental illnesses is imperative during residency training in Graduate Medical Education (GME) programs and throughout professional practice. We hope that this cross-sectional study will cultivate mindfulness and improve the healthcare outcomes of stigmatized individuals.

Methodology

A cross-sectional study using a web-based Attitude to Mental Illness Questionnaire (AMIQ) with additional scenarios was utilized to assess stigma among resident physicians in differing specialties in a hospital training system. The research investigators collected anonymous data on demographics, characteristics, specialties, and the AMIQ. In eight vignettes, participants responded to five items using a five-point Likert scale assessing attitudes toward individuals.

Results

Of the 104 resident physicians enrolled in the hospital training system where the study was conducted, 58 (56%) volunteered to participate. The participating residents markedly exhibited negative attitudes toward the individual in the vignette with multiple inpatient psychiatric admissions. Residents also exhibited more negative attitudes toward individuals with schizophrenia, self-harm by way of overdose, psychedelic users, cannabis users, and alcohol problems compared to diabetic and Christian individuals.

Conclusions

Residents exhibited negative attitudes toward individuals with mental illness. Additional research would further our understanding of the reasons for physician bias. Moreover, GME programs and medical educators can play a crucial role in mitigating stigma among future physicians, thereby enhancing care for individuals with mental illness.

## Introduction

The term *stigma* originated from the Greek *stizein* meaning *to tattoo* and was used in early English, referred to as a scar left by a hot iron or brand [1]. Today, this scar is symbolic, referring to society’s negative beliefs about groups of people [1]. It is well documented that patients with mental illness experience this stigma in the form of prejudice and ostracization. Mental illness conditions such as schizophrenia and depression pose significant challenges for affected individuals, not only due to the disorder but also experienced stigmatization itself.

There is, unfortunately, pervasive stigmatization that individuals with mental illness encounter within healthcare settings. Extensive studies performed on the attitudes of physicians toward individuals with mental illnesses such as schizophrenia have shown that there is a perceived dangerousness, a desire for social distance, and poor attitudes [2-4]. The perception that individuals with schizophrenia are more likely to be dangerous to others is a recurrent finding. Physicians also perceived mentally ill patients as time-intensive and potentially noncompliant with treatment [2,3,5]. Additionally, doubts about the ability of individuals with schizophrenia to self-manage health [2,4] further highlight physician attitudes toward individuals with mental illness.

Physician attitudes and bias result in mentally ill individuals receiving substandard healthcare. Many such individuals report feeling "devalued, dismissed, and dehumanized" [6,7] by healthcare professionals. Individuals with mental illness who also suffer from physical conditions such as cardiovascular disease, respiratory illness, and diabetes have been observed to have a lifespan that is 10 to 20 years shorter compared to those without mental illness. This disparity is likely due to their somatic complaints not being addressed as effectively as those of individuals without mental illness. Despite having twice as many healthcare interactions, mentally ill individuals undergo fewer physical check-ups, receive fewer screenings, are prescribed fewer medications, and undergo fewer procedures [8,12,13]. Additionally, they are less likely to be diagnosed with cardiovascular and cancer conditions [8,12]. Hospitalization for medical care among individuals with mental illness is often associated with poorer outcomes, including more adverse events, prolonged stays in intensive care units, and increased complications compared to those without mental illness [8,14,15], suggesting a deficiency in the healthcare system’s ability to prevent, identify, and treat physical diseases during hospitalization for a mental disorder.

Recognizing stigmatization toward people with mental illnesses will help improve deficiencies in the care these individuals receive. Raising awareness of stigmatization among resident physicians will improve the quality of physical and psychiatric healthcare administered to patients. Addressing stigma toward patients with mental illnesses will affect the residents’ quality of care and effectiveness as physicians throughout and beyond training.

## Materials and methods

This cross-sectional study was performed among multiple Graduate Medical Education (GME) specialty training programs for resident physicians in Emergency Medicine (EM), Family Medicine (FM), Internal Medicine (IM), Psychiatry (PM), and Transitional Year (TY) programs within a singular hospital training system. Per inclusion criteria, any resident physician enrolled in the hospital's training program was eligible for participation, while nonresidents and residents not within the program were excluded. All resident physicians within this program were invited to participate. The investigators obtained informed consent from those who volunteered to participate. Demographic and characteristic data were collected using online survey software and compiled confidentially and anonymously. Survey data were collected over one week.

The survey software administered the Attitudes Toward Mental Illness Questionnaire (AMIQ), a self-administered questionnaire measuring one’s attitude toward mental illness. The AMIQ was first developed by Cunningham et al. [16,17] to simplify stigmatization assessment [18] and was then later validated by Luty et al. [16,18]. This validation determined the AMIQ to be a reliable psychometric instrument with good stability and test-retest reliability, making it helpful in various mental health stigma research settings [18]. For this study, one vignette was slightly modified, substituting acetaminophen for the word paracetamol for better regional understanding. Additional vignettes to assess stigmatization toward marijuana and psychedelic use were also added to evaluate bias toward other substance use populations.

The participating residents were required to read a vignette of a fictional individual before responding to 5 items that would assess attitude levels (Appendix). These five items were the same for each vignette. The vignettes were presented to all residents in the following order and describe fictional individuals with various backgrounds: an individual (Michael) with schizophrenia, an individual (Steve) who is a recovering alcoholic, an individual (Michael) with a history of multiple psychiatric hospitalizations, an individual (John) currently smoking cannabis, an individual (Michael) currently using psychedelics, an individual (Peter) with diabetes, an individual (Tim) with a history of overdose/self-harm, and an individual (Steve) who practices Christianity. The AMIQ includes vignettes of a diabetic and Christian to serve as control as these are nonstigmatized individuals [18]. The participants were informed that each vignette is about a distinct individual despite some having the same names. The participants were required to answer each item with responses ranging from very unlikely/strongly disagree to very likely/strongly agree (Appendix). Answers were based on a five-point Likert scale (-2 to +2), with negative scoring assigned to negative attitude and positive scoring for positive attitude. For each vignette, participants’ scores for the five items were added, with the total score ranging from -10 to +10.

Statistical software R version 4.3.3 was used to compute descriptive statistics and perform the Wilcoxon-Rank-Sum test. The study was conducted at a community hospital lacking an Institutional Review Board (IRB). Consequently, it underwent review at Harding University’s IRB, where it was deemed exempt from IRB oversight.

## Results

Of the 104 residents enrolled in the hospital training system who were invited to participate in the study, 58 (56%) agreed to participate. Their demographic and characteristic data are detailed in Table 1. They represented various medical specialties, with 11 from FM, 15 from EM, 15 from IM, 16 from PM, and six from TY. Thirty-nine (67%) were male, and 19 (33%) were female. Approximately half of the participants (53%) were not close to someone diagnosed with a psychiatric disorder.

**Table 1:** Demographics and characteristics. ^*^Psychiatric disorders include schizophrenia, psychosis, bipolar 1 or 2, generalized anxiety disorder, panic disorder, obsessive-compulsive disorder, substance dependence, suicidal ideation, or previous hospitalization due to self-harm thoughts.

Description	Answer choices	N	%
Sex	Male	39	67%
	Female	19	33%
Age	Mean ± SD	31 ± 4.1	
Ethnicity	Caucasian	31	54%
	African American	2	4%
	Latin or Hispanic	5	9%
	Asian	16	28%
	Prefer not to say	4	7%
Current program	Emergency Medicine	14	24%
	Family Medicine	8	14%
	Internal Medicine	14	24%
	Psychiatry	16	28%
	Transitional Year	6	10%
Postgraduate Year (PGY)	PGY-1	22	38%
	PGY-2	16	28%
	PGY-3	17	29%
	PGY-4	3	5%
Have you ever been told that you have a psychiatric disorder?^*^	Yes	9	16%
	No	43	74%
	Prefer not to say	6	10%
Are you close with anyone who has a psychiatric disorder?^*^	Yes	31	53%
	No	26	45%
	Prefer not to say	1	2%

**Figure 1:**
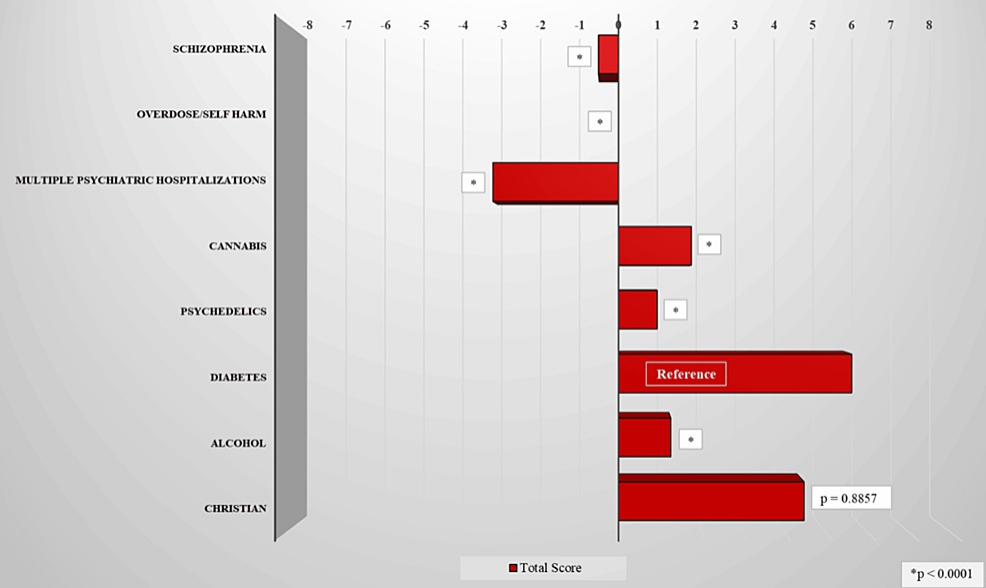
Total score across all participating residents stratified by vignette.

The most positive attitude levels were toward the individual with diabetes, the average Likert score of 5.6 (mean [M] = 5.6, standard deviation [SD] = 3.7), and the practicing Christian (M = 4.5, SD = 3.7). Compared to the diabetes vignette, the more negative attitudes were primarily directed toward the individual with multiple psychiatric inpatient hospitalizations (M = -3.02, SD = 4.12), followed by the schizophrenic (M = -0.48, SD = 3.07), the individual with self-harm (M = 0, SD = 3.30), the psychedelic user (M = 0.93, SD = 4.60), the recovering alcoholic (M = 1.26, SD = 3.82), and the cannabis user (M = 1.75, SD = 3.16) (Table 2 and Figure 1).

**Table 2:** Vignettes and total scores across all participating residents.

Vignettes	Category	Total score		*P*-value
		Mean	SD	
Michael has schizophrenia. He needs an injection of medication every two weeks. He was detained in a hospital for several weeks two years ago because he was hearing voices from the devil and thought he had power to cause earthquakes.	Schizophrenia	-0.48	3.07	<0.0001
Steve has been drinking heavily for five years. He is now going for treatment and has started attending Alcoholics Anonymous meetings.	Alcohol	1.26	3.82	<0.0001
Michael has been hospitalized for the sixth time for his psychiatric diagnosis.	Multiple psychiatric hospitalizations	-3.02	4.12	<0.0001
John has been smoking cannabis daily for five years.	Cannabis	1.75	3.16	<0.0001
Michael has been using psychedelics monthly for one year.	Psychedelics	0.93	4.6	<0.0001
Peter has diabetes and needs to inject insulin every day. (Reference)	Diabetes	5.6	3.7	1
Tim is depressed and took an acetaminophen overdose last month to try and hurt himself.	Overdose/self-harm	0	3.3	<0.0001
Steve is a practicing Christian. He attends church every Sunday and attempts to lead a Christian life.	Christian	4.46	4.26	0.8857

**Figure 2:**
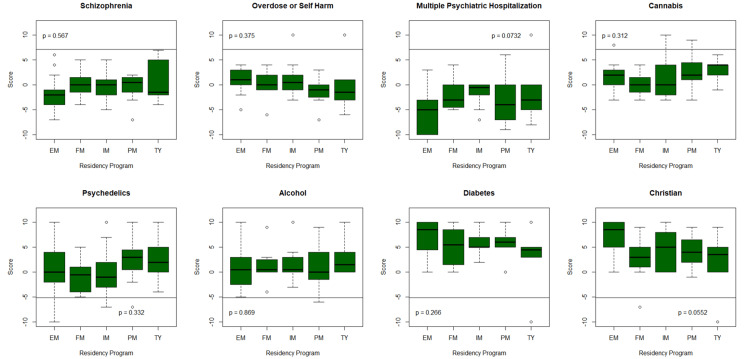
Comparison of total scores among the residency programs. Residency programs surveyed include Emergency Medicine (EM), Family Medicine (FM), Internal Medicine (IM), Psychiatry (PM), and Transitional Year (TY).

Residents' attitudes in different specialties and PGY, as well as whether they have been diagnosed with or are close to someone who has been diagnosed with a psychiatric disorder, were also compared in each corresponding vignette (Table 3; Figure 2). Regarding the individual with schizophrenia, TY residents were the most positive in attitude, while EM residents were the most negative. For the individual with self-harm, both EM and FM residents were the most positive in attitude, while PM residents were the most negative. For the individuals with multiple psychiatric hospitalizations, both IM and TY residents were the most positive in attitude, with EM residents being the most negative. For the cannabis users, TY residents were the most positive and FM residents were the most negative. PM residents were the most positive toward the psychedelic user, and FM residents were the most negative. Finally, for the recovering alcoholic, TY residents were the most positive, while EM residents were the most negative.

**Table 3:** Scoring by specialty, postgraduate year, and familiarity with psychiatric disorder for each vignette. *Psychiatric disorders include schizophrenia, psychosis, bipolar 1 or 2, generalized anxiety disorder, panic disorder, obsessive-compulsive disorder, substance dependence, suicidal ideation, or previous hospitalization due to self-harm thoughts.

Description	N	%	Vignette																							
			Schizophrenia			Overdose/self-harm			Multiple psychiatric hospitalizations			Cannabis			Psychedelics			Alcohol			Diabetes (Reference)			Christian		
			Mean	± SD	*P*-value	Mean	± SD	*P*-value	Mean	± SD	*P*-value	Mean	± SD	*P*-value	Mean	± SD	*P*-value	Mean	± SD	*P*-value	Mean	± SD	*P*-value	Mean	± SD	P-value
Specialty																										
Emergency Medicine	14	24%	-1.64	3.61	0.0001	0.79	2.46	0.00040	-5.50	4.07	0.00003	1.69	3.01	0.0018	0.69	5.57	0.0040	0.75	4.16	0.0028	7.00	3.46	1.00	7.17	3.19	0.9529
Family Medicine	8	14%	0.13	2.70	0.0151	0.00	2.98	0.01729	-2.00	3.12	0.0037	0.13	2.23	0.0131	-0.88	3.40	0.0096	1.38	3.70	0.0714	5.13	3.87	1.00	2.50	4.69	0.3428
Internal Medicine	14	24%	-0.28	2.79	<0.00001	0.79	3.42	0.00034	-1.50	2.14	0.00001	1.21	3.70	0.0020	-0.21	4.53	0.0011	1.21	3.17	0.0004	5.71	2.49	1.00	4.71	3.97	0.4839
Psychiatry	16	28%	-0.31	2.47	0.0001	-1.40	2.92	0.00002	-3.26	4.38	0.00004	2.60	3.27	0.0153	2.53	4.09	0.0171	1.00	4.42	0.0036	5.73	2.94	1.00	4.13	3.11	0.1058
Transitional year	6	10%	0.50	4.42	0.4673	-0.16	5.49	0.2607	-1.50	6.25	0.2607	3.20	2.40	0.6242	2.50	4.76	0.5711	2.83	3.92	0.4167	2.83	6.74	1.00	1.83	6.49	0.6267
Training year																										
PGY-1	22	38%	0.23	3.44	0.0003	0.18	3.20	0.0001	-1.68	4.78	0.0001	2.50	3.32	0.0182	1.45	5.24	0.0171	2.52	3.70	0.0188	4.95	4.82	1.00	3.62	4.92	0.2588
PGY-2	16	28%	-0.63	2.42	0.00004	0.31	3.48	0.0003	-3.19	2.69	<0.00001	1.31	3.44	0.0011	0.63	4.13	0.0007	1.00	3.27	0.0006	6.19	3.51	1.00	4.75	4.82	0.4464
PGY-3 and 4	20	34%	-1.15	3.10	<0.00001	-0.47	3.39	<0.00001	-4.42	3.96	<0.00001	1.22	2.65	0.00002	0.56	4.36	0.00013	0.00	4.13	0.00008	5.83	2.07	1.00	5.17	2.66	0.2925
Diagnosed with a psychiatric disorder*																										
Yes	9	17%	-0.78	3.96	0.0032	-0.67	3.39	0.0012	-4.22	4.68	0.0007	3.33	3.00	0.0403	2.67	5.00	0.0493	0.67	5.34	0.0129	6.89	3.41	1.00	5.89	4.01	0.5281
No	43	83%	-0.56	2.83	<0.00001	-0.12	3.13	<0.00001	-3.26	3.65	<0.00001	1.43	3.12	<0.00001	0.21	4.27	<0.00001	1.32	3.33	<0.00001	5.80	2.91	1.00	5.00	3.32	0.1822
Close with anyone diagnosed with a psychiatric disorder*																										
Yes	31	54%	-0.19	3.23	<0.00001	0.57	2.96	<0.00001	-2.17	4.62	<0.00001	2.86	2.71	0.0018	2.10	4.07	0.0015	1.04	4.44	0.0001	5.00	4.20	1.00	3.82	4.78	0.1651
No	26	46%	-0.69	2.90	<0.00001	-0.63	3.67	<0.00001	-3.73	3.13	<0.00001	0.54	3.28	<0.00001	-0.35	4.97	<0.00001	1.38	3.13	<0.00001	6.08	3.01	1.00	4.92	3.54	0.3565

The TY residents displayed more positive attitudes in most vignettes toward individuals with mental illness or substance abuse. EM residents displayed greater negative attitudes toward individuals with mental illness, and FM residents displayed greater negative attitudes toward individuals with substance use. Notably, PM residents also demonstrated negative attitudes toward individuals with mental illness but were more positive toward individuals with substance use issues (Table 3; Figure 2).

The analysis of attitude levels by training year could not include residents in their fourth year as there were only three PGY-4 participants, all from the psychiatry specialty, which limited our comparisons of residents up to PGY-3. As residents were further along in their training, their attitudes toward individuals with mental illness or substance abuse tended to become more negative. PGY-2 residents showed some exceptions to this trend by displaying a more positive attitude toward the individual who self-harmed by overdose but a more negative attitude toward the cannabis user. Conversely, residents in more advanced stages of training tended to have more positive attitude levels toward individuals identified as Christian and diabetic.

Interestingly, residents close to those diagnosed with a psychiatric disorder had more positive attitudes toward individuals with mental illness, cannabis use, and psychedelic use (Table 3). Conversely, residents not close to anyone diagnosed with a psychiatric disorder had more positive attitudes toward the recovering alcoholic, diabetic, and Christian.

In summary, this study revealed a spectrum of attitudes among resident physicians toward individuals with mental illness and substance abuse. Positive attitudes were expectedly most pronounced toward individuals with diabetes and practicing Christians per the AMIQ survey design, while individuals with multiple psychiatric hospitalizations and schizophrenia elicited comparably more negative attitudes. Specialty-wise, TY residents had more positive attitudes toward individuals in most scenarios, while EM residents were more negative toward individuals with mental illness and FM residents were more negative toward substance users. Attitudes varied by training year, with residents displaying increasingly negative attitudes toward individuals with mentally illness, the further along the training was.

## Discussion

Our findings align with established studies on physician stigma, confirming the presence of stigmatized perspectives among physicians in training across diverse specialties toward individuals with mental illnesses. Resident physicians were overwhelmingly negative in attitude toward the individual with multiple psychiatric hospitalizations as well as schizophrenia. Resolving the prevalent negative attitudes toward individuals with mental illness poses a significant challenge, but education and familiarity with mental illness will likely contribute to improvement in attitude levels toward these stigmatized individuals. This is supported by our data that demonstrate that familiarity with mental illness is associated with more positive attitudes among residents close to individuals with mental illness.

While this study gives us important insights, there are limitations. This cross-sectional study exhibits a notable degree of selection bias. Participation therein was voluntary, leading to a participation rate of merely 56% among residents enrolled in the training program. Consequently, this study’s observed traits and biases need to be more completely representative across the resident population at this hospital training program. In addition, our sample size was not large, being exclusively resident physicians affiliated with a singular hospital training program, which would likely result in not being indicative of attitude levels among resident physicians nationwide. The sample size was also lacking for transitional year and family medicine residents. Transitional year residents who volunteered were all PGY-1 residents; their backgrounds and future specialties were also not inquired, further contributing to variance in this study. Additionally, we did not account for external factors that may have contributed to negative attitudes among resident physicians, such as stress due to more difficult rotations or not yet having exposure to psychiatric rotations. It is still unclear whether worsening attitudes among upper-level residents are the result of repeated exposure to patients with mental illness or simply due to these upper-level residents receiving inadequate training. Following the lower-level residents each year to perform repeat assessments of attitude levels could overcome this limitation. Future research could explore reasons for physician bias in addition to obtaining a larger sample of resident physicians across multiple nationwide training programs.

To reduce stigma toward patients with psychiatric disorders, GME program directors and medical educators could consider stigma reduction curricula to raise awareness of physician bias toward these stigmatized patient populations. The cause of negative attitudes and bias were not inquired of participants of this study, but a common physician perception is that individuals with schizophrenia are more prone to violence. An essential step toward mitigating this perception would involve educating physicians about specific symptoms within mental illnesses that correlate with violent behavior rather than solely attributing the risk of violence to the mental illness diagnosis itself [19,20]. By equipping physicians with the knowledge to identify these symptomatic indicators of potential danger in patients, the overall quality of care for individuals with mental illness can be enhanced. Recognizing patient features/symptoms indicative of danger to healthcare staff [20] would help residents be less weary and, thus, less biased in their treatment of these populations.

## Conclusions

In summary, this study highlights the ongoing stigmatization and bias among resident physicians toward populations with mental illness and substance use. These results emphasize the need for improvements in medical education to improve attitudes toward stigmatized patient populations. Familiarity with mental illness across all specialties resulted in less stigmatization, supporting our belief that by fostering understanding and interdisciplinary collaboration, GME training programs, and medical educators can play a significant role in reducing stigmatization among healthcare providers, improving the quality of care for individuals with mental health concerns.

## Appendices

Appendix

Attitudes to Mental Illness Questionnaire (AMIQ) format

Vignette 1:* Michael has schizophrenia. He needs an injection of medication every 2 weeks.  He was detained in a hospital for several weeks 2 years ago because he was hearing voices from the devil and thought he had power to cause earthquakes. *

1. Do you think that this would damage Michael's career?

Strongly agree (-2), Agree (-1), Neutral (0), Disagree (+1), Strongly disagree (+2), Don’t know (0).

2. I would be comfortable if Michael was my colleague at work.

Strongly agree (+2), Agree (+1), Neutral (0), Disagree (-1), Strongly disagree (-2), Don’t know (0).

3. I would be comfortable about inviting Michael to a dinner party.

Strongly agree (+2), Agree (+1), Neutral (0), Disagree (-1), Strongly disagree (-2), Don’t know (0).

4. How likely do you think it would be for Michael's wife to leave him?

Very likely (-2), Quite likely (-1), Neutral (0), Unlikely (+1), Very unlikely (+2), Don’t know (0).

5. How likely do you think it would be for Michael to get in trouble with the law?

Very likely (-2), Quite likely (-1), Neutral (0), Unlikely (+1), Very unlikely (+2), Don’t know (0).

## References

[1] (2024). “Stigma.” Merriam-Webster.com Dictionary. https://www.merriam-webster.com/dictionary/stigma.

[2] Stone EM, Chen LN, Daumit GL, Linden S, McGinty EE (2019). General medical clinicians’ attitudes toward people with serious mental illness: a scoping review. J Behav Health Serv Res.

[3] Lawrie SM, Martin K, McNeill G (1998). General practitioners' attitudes to psychiatric and medical illness. Psychol Med.

[4] Magliano L, Punzo R, Strino A, Acone R, Affuso G, Read J (2017). General practitioners' beliefs about people with schizophrenia and whether they should be subject to discriminatory treatment when in medical hospital: The mediating role of dangerousness perception. Am J Orthopsychiatry.

[5] Lam TP, Lam KF, Lam EW, Ku YS (2013). Attitudes of primary care physicians towards patients with mental illness in Hong Kong. Asia Pac Psychiatry.

[6] Knaak S, Mantler E, Szeto A (2017). Mental illness-related stigma in healthcare: barriers to access and care and evidence-based solutions. Healthc Manage Forum.

[7] Hamilton S, Pinfold V, Cotney J (2016). Qualitative analysis of mental health service users' reported experiences of discrimination. Acta Psychiatr Scand.

[8] Liu NH, Daumit GL, Dua T (2017). Excess mortality in persons with severe mental disorders: a multilevel intervention framework and priorities for clinical practice, policy and research agendas. World Psychiatry.

[9] Wahlbeck K, Westman J, Nordentoft M, Gissler M, Laursen TM (2011). Outcomes of Nordic mental health systems: life expectancy of patients with mental disorders. Br J Psychiatry.

[10] Laursen TM, Musliner KL, Benros ME, Vestergaard M, Munk-Olsen T (2016). Mortality and life expectancy in persons with severe unipolar depression. J Affect Disord.

[11] Saha S, Chant D, McGrath J (2007). A systematic review of mortality in schizophrenia: is the differential mortality gap worsening over time?. Arch Gen Psychiatry.

[12] Lawrence DM, Holman CD, Jablensky AV, Hobbs MS (2003). Death rate from ischaemic heart disease in Western Australian psychiatric patients 1980-1998. Br J Psychiatry.

[13] Kisely S, Campbell LA, Wang Y (2009). Treatment of ischaemic heart disease and stroke in individuals with psychosis under universal healthcare. Br J Psychiatry.

[14] Chen YH, Lin HC, Lin HC (2011). Poor clinical outcomes among pneumonia patients with schizophrenia. Schizophr Bull.

[15] Daumit GL, Pronovost PJ, Anthony CB, Guallar E, Steinwachs DM, Ford DE (2006). Adverse events during medical and surgical hospitalizations for persons with schizophrenia. Arch Gen Psychiatry.

[16] Stacchini L, Fonzo M, Catalini A (2024). An Italian validation of the 5-item attitudes to mental illness questionnaire (AMIQ): a useful tool for rapid assessment of stigma, acceptance, and tolerance. Healthcare (Basel).

[17] Cunningham JA, Sobell LC, Chow VM (1993). What's in a label? The effects of substance types and labels on treatment considerations and stigma. J Stud Alcohol.

[18] Luty J, Fekadu D, Umoh O, Gallagher J (2006). Validation of a short instrument to measure stigmatised attitudes towards mental illness. Psychiatr Bull.

[19] DeAngelis DeAngelis, T T (2022). Mental illness and violence: debunking myths, addressing realities. Monitor Psychol.

[20] Peterson JK, Skeem J, Kennealy P, Bray B, Zvonkovic A (2014). How often and how consistently do symptoms directly precede criminal behavior among offenders with mental illness?. Law Hum Behav.

